# On the Therapeutic Action of Aconite and Its Preparations

**Published:** 1869-09

**Authors:** Sidney Ringer

**Affiliations:** Prof. of Therapeutics at University College, and Physician to University College Hospital


On the Therapeutic Action of Aconite and its Preparations.—By Dr. Sidney Ringer, Prof. of Therapeutics at University College, and Physician to University College Hospital.—Of all the drugs we possess there are certainly none more valuable than aconite. Its virtues by most persons are only beginning to be appreciated, but it is not difficult to foresee that in a short time it will be most extensively employed in the diseases immediately to be noticed.

As external applications, the liniment or ointment is used to relieve pain. They appear to possess power over pain of different kinds. In the neuralgias of the brow or face these applications are sometimes of the greatest use, and often relieve, either permanently or temporarily, the distressing pain of these complaints. But while in many instances their effects are immediate and permanent, yet it must be confessed that in the majority of cases the pain is unaffected. Neither can we, with our present knowledge, predict with any certainty the cases in which the application will be useful, or those in which it will fail. This much, however, is ascertained, that those neuralgias which depend on decayed teeth or diseased bone, or on tumors pressing on the nerves, are beyond the control of aconite. But these are not the only forms of neuralgia over which aconite can not prevail. No doubt in some instances the failure can be explained by the badness of the preparation; but even when this is excellent it is not uncommon for the pain to remain unabated after the application. As, however, no harm can possibly follow their employment, they should always be tried; and if unsuccessful, then resource can be had to other modes of treatment. If aconite succeeds at all, it will succeed at once; and hence, if no relief is speedily obtained, it is useless to continue its employment. The preparation should be sufficiently strong to produce decided numbness and tingling in the skin over which it is rubbed; and where of service, these sensations replace the pain, which does not return when the effects of the aconite have worn off.

The ointment and liniment should be applied with friction, which very greatly heightens their activity. Of the ointment, a piece the size of a pea or bean should be rubbed into the skin till it has disappeared. Care should be taken while using these powerful and poisonous applications that they are not rubbed into wounds or cracks of the skin, nor brought into contact with absorbent tissues, such as mucous membranes and the conjunctiva of the eye. Spinal irritation and intercostal neuralgia may in many instances be removed by aconite ointment, and sciatica sometimes yields to the same remedy. The former complaints are better treated by the belladonna preparations.

It is on account of its power to control inflammation and subdue the accompanying fever that aconite is to be most esteemed. The power of this drug over inflammation is little less than marvellous. It can sometimes at once cut short the inflammation. It does not remove the products of inflammation when these are formed, but by controlling the disease, it prevents the formation of these, and so saves the tissues from further injury. It is, therefore, in the early stage of inflammation that the good effects of this plant are most conspicuous; still, although the disease may have progressed to some extent, and have injured the organs by the formation of new and diseased products, while the inflammation is extending aconite does good. It is useful wherever there is acute inflammation of any tissues of the body. The good it accomplishes can be shown both by the amelioration of the symptoms, and, still better, by the changes it effects in the inflamed tissues when these are visible, as in pharyngitis, tonsillitis, &c.

As might be expected, the results of aconite are most apparent when the inflammation is not extensive, or not very severe, as in the catarrh of children, or in tonsillitis, or in acute sore-throat. In these comparatively mild diseases, especially if the aconite be given in the earliest stage of the inflammation, when the chill is still on the patient, the following consequences will very generally be witnessed:—In a few hours the skin, which before was dry, hot and burning, becomes comfortably moist; and in a little time longer, it is bathed in a profuse perspiration, which may be so great that drops of sweat run down the face and chest. With this appearance of sweat many of the distressing sensations—such as the restlessness, chilliness, or heat and dryness of the skin—are removed. At the same time the quickened pulse is much reduced in frequency, and in a period of twenty-four to forty-eight hours, it and the temperature have reached their natural state. It is rare that a quinsy or acute sore-throat, if caught at the commencement, can not be disposed of in twenty-four to forty-eight hours. The sweating may continue for a few days after the decline of the fever on slight provocations; but it then ceases.

The appearance of the inflamed part also exhibits, in a striking degree, the beneficial effects of the drug. Thus large livid, red, glazed, and dry tonsils may often in twenty-four hours have their appearance completely altered. If the medicine has been given before much lymph has been formed in these organs, in the time named the swelling and most of the redness will have disappeared; and the mucous membrane will have that look which proves the acute inflammation to have subsided—namely, it has become moist, and is bathed with mucus or pus. If just at this stage some strong astringent—such as glycerine of tannin—be applied, most of the remaining diseased appearance of the pain, if it continues, will be removed. Such are the visible effects of aconite on inflamed tonsils, &c.

